# Dogs distinguish human intentional and unintentional action

**DOI:** 10.1038/s41598-021-94374-3

**Published:** 2021-09-01

**Authors:** Britta Schünemann, Judith Keller, Hannes Rakoczy, Tanya Behne, Juliane Bräuer

**Affiliations:** 1 Department of Developmental Psychology, University of Göttingen, Waldweg 26, 37073 Göttingen, Germany; 2 Department of Biology, University of Hamburg, Martin-Luther-King-Platz 3, 20146 Hamburg, Germany; 3 Department of Linguistic and Cultural Evolution, Max Planck Institute for the Science of Human History, Kahlaische Strasse 10, 07745 Jena, Germany; 4 Department for General Psychology and Cognitive Neuroscience, Friedrich Schiller University of Jena, Am Steiger 3, 07743 Jena, Germany

**Keywords:** Social evolution, Animal behaviour

## Abstract

When dogs interact with humans, they often show appropriate reactions to human intentional action. But it is unclear from these everyday observations whether the dogs simply respond to the action outcomes or whether they are able to discriminate between different categories of actions. Are dogs able to distinguish intentional human actions from unintentional ones, even when the action outcomes are the same? We tested dogs’ ability to discriminate these action categories by adapting the so-called “Unwilling vs. Unable” paradigm. This paradigm compares subjects’ reactions to intentional and unintentional human behaviour. All dogs received three conditions: In the unwilling-condition, an experimenter intentionally withheld a reward from them. In the two unable-conditions, she unintentionally withheld the reward, either because she was clumsy or because she was physically prevented from giving the reward to the dog. Dogs clearly distinguished in their spontaneous behaviour between unwilling- and unable-conditions. This indicates that dogs indeed distinguish intentional actions from unintentional behaviour. We critically discuss our findings with regard to dogs’ understanding of human intentional action.

## Introduction

When humans interact with other humans, this interaction depends to a substantial extent on ascribing intentions to one another. We could hardly make sense of other agents’ actions if we could not consider what the agent planned and whether he or she acted intentionally or accidentally^1,2^. The concept of intention is a central part of our Theory of Mind, the ability to attribute mental states to others and ourselves^3^. Theory of Mind has long been regarded as a uniquely human ability. However, while this may be true for our full-fledged adult Theory of Mind that includes the ascription of complex subjective states, accumulating evidence suggests that certain basic capacities to ascribe simple mental states to other agents are present in some non-human species, such as apes and birds. For example, chimpanzees track whether other agents can or cannot see a given scene^4,5^, and male Eurasian jays react appropriately to a females’ desires even if they do not share these desires^6^ (for an overview see^7–9^). In human ontogeny, the ascription of intentions is one of the primary forms of Theory of Mind: Even infants understand actions as goal-directed^10^, differentiate intentional from unintentional behaviour^11,12^, and rationally imitate failed actions^13^.

But humans are not the only ones who rely substantially on making sense of human actions. The dog is probably the animal whose history and everyday life are most interwoven with that of humans. Having evolved in close proximity to humans, dogs have developed special skills for forming close social bonds with us^14–16^. In this close relationship, they are confronted with different forms of human intentionality on a regular basis, for instance communicative intentions, when humans either intend or do not intend to communicate with them using certain behavioural and vocal cues^17^. Also, dogs experience how humans direct their actions to certain goals, and encounter intentional and unintentional actions, for instance, when humans lie on the grass intentionally versus when they trip.

But do dogs show appropriate responses to human intentional actions because they are simply responding to an action’s environmental outcome or because they actually recognize the human’s intention? In cases where an intention has been realized successfully it is hard to tell whether dogs react to the action’s outcome or to the underlying intention. Accordingly, the methodological approach to study intentions in both human and non-human animals has been to examine their responses to failed attempts and accidental behavior^12,18–23^. To our knowledge this approach has not been used with dogs, yet.

There is ample research on dogs’ capacity to react appropriately to humans’ mental states: Dogs register a human’s attentive state when they decide whether to steal food^24^, to beg for food^25^, or to obey commands^26^. To some degree, they even seem to be able to take a human’s visual perspective^27–30^. Moreover, dogs outperform even chimpanzees in reacting appropriately to human pointing gestures^31–36^, and attend to the referential nature of the human’s gaze during social interactions^17,37,38^, as well as to the communicative intent of the human^17^. They even take contextual information into account rather than blindly following a pointing gesture^39,40^. For instance, they understand that a human does not intend to communicate something to them when that person looks at their watch and thereby inadvertently points towards a certain location^17^.

However, when it comes to dogs’ capacity for ascribing goals, the evidence is mixed. At first glance, some evidence speaks in favour of such a capacity. Dogs expect agents to keep acting towards a certain goal even if that requires a change of action^41^. Also, dogs appear to recognize the target of a human’s search and show informative motives to help find a hidden object^42^. Furthermore, dogs consider an agent’s goal when they rationally imitate the agent’s actions^43,44^. For example, when dogs observed another dog that used his/her paw to pull a rod, because his/her mouth was occupied holding a ball, they used their mouth to pull the rod (rational action). In contrast, when they observed that the dog used his/her paw to pull the rod for no visible reason, they imitated this inefficient action. However, upon closer examination, dogs’ performance in these tasks can be explained by low-level, submentalizing explanations (domain-general cognitive processes that may appear like true mentalizing but turn out not to be upon closer inspection)^45,46^. Dogs seem to have performed the rational action in the first condition only because they were distracted by the presence of a ball and simply missed the presented inefficient action^47^. Moreover, dogs show difficulties in recognizing humans’ implicit goals. They only help if the human explicitly communicates her goals^36,42^, or if the goal is attractive to them^48^.

Thus, while dogs appear to understand humans’ communicative intentions, their concept of human goals appears to be based much more on trial-and-error learning. Accordingly, it may seem unlikely that dogs have a grasp of human intentions^36,49,50^^.^ However, intentions have different guises. We commonly distinguish between prior intentions (directed at the future and related to planning) and intentions-in-action (directed at actions in the here and now)^2,51^. Accordingly, it is possible that their notion of human intentions is not all-or-nothing, but rather comprises (only) some aspects of understanding, presumably those that have been of direct importance to them. Prior intentions are future-directed intentions that commit the agent to an action, such as: “I will walk the dog tomorrow”. It is sometimes doubted, however, that non-human animals can even hold prior intentions themselves, let alone ascribe them to other agents^2,52^. However, what is more important in interactions is to make sense of intentions-in-action. Intentions-in-action are present-directed intentions (“I’m walking the dog now”) that underlie the voluntary guidance of action. Observing actions enables an observer to interpret actions as intentional (“she lay down in the grass willingly”) and distinguish it from unintentional behaviour (“she tripped over a root”). It appears much more likely that dogs recognize this basic form of intention. But do they really?

A very useful non-verbal approach to test for such a capacity is the so-called *“Unwilling vs. Unable”* paradigm. This approach refrains from imitations. Instead, it examines whether subjects react differently towards a human agent who either intentionally (being unwilling) or unintentionally (being unable) withheld rewards from them. Using this paradigm, the capacity to distinguish intentional from unintentional behaviour has been found not only in human infants^12,20^ but also in chimpanzees^18^, African grey parrots^21^, capuchins^22^, Tonkean macaques^19^ and horses^23^. Some of these species lost interest in the unwilling-condition earlier than in the unable-condition. Other species were found to differentiate in their begging or aggressive behaviour, or in their vocalizations (for a detailed overview of measured behavioural reactions see Supplementary Material). From an evolutionary point of view, it is interesting that even undomesticated species appear to recognize intention-in-action of humans. It suggests that such a capacity is present for interactions with other individuals and can then be generalized flexibly to interaction with humans. However, it is also possible that ontogeny plays a role here, i.e., that these tested captive animals had the chance to learn this skill in their interactions with humans. For dogs, we expect that there is both, a potential selection pressure during domestication to discriminate human intentions, but also the extensive possibility to learn this during ontogeny.

**Figure 1:**
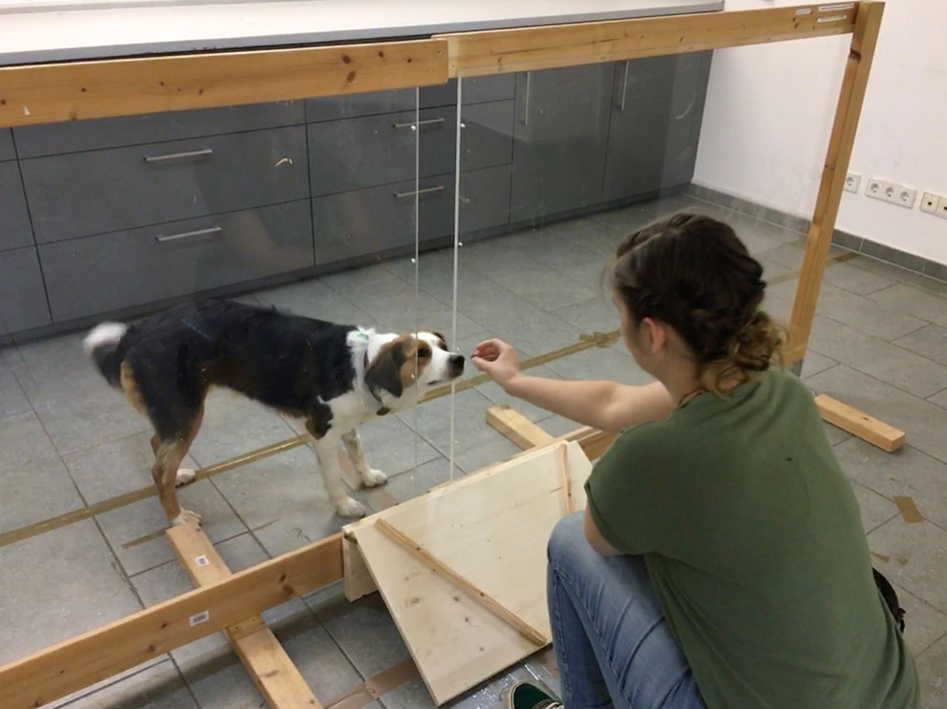
Experimental set-up with opened gap.

To explore dogs’ ability to distinguish intentional from unintentional actions, the current study adapted the *“Unwilling vs. Unable”* paradigm to make it suitable for dogs: The dogs (*N* = 51) were separated from the experimenter by a transparent partition wall (Fig. 1). The experimenter administered rewards to the dog through a gap in the partition. In the unwilling*-*condition, the experimenter suddenly withdrew the reward from the dog with an intentional movement and placed it in front of herself. In the unable-clumsy condition, the experimenter pretended to try to administer the reward, but the reward “accidentally” fell out of her hand before she could pass it through the gap. Similarly, in the unable-blocked condition, she tried to administer the reward but was unable to pass it through the gap because it was blocked. In all three conditions, the experimenter left the rewards on the floor in front of her (on her side of the partition) after failing to administer them. The dogs received all three conditions in a counterbalanced order (see Supplementary Material for an overview of orders). Thus, all three conditions are similar in that a reward is brought near the dog, but then is never passed through the gap. They are only different in whether this action was performed in an intentional manner or in an unintentional manner, because some circumstance hindered the agent to perform the action. If dogs are indeed able to ascribe intention-in-action to humans, we would expect them to show different reactions in the unwilling-condition compared to the two unable-conditions. As our primary measure, we looked at dogs’ waiting behaviour: How long do the dogs wait before they go around the partition and approach the reward? We predicted that if dogs are able to identify human intentional action, they should wait longer to approach the reward in the unwilling-condition than in the two unable-conditions. The logic behind this was the following: When the experimenter intentionally withholds the reward, the dogs should hesitate to approach it (because they predict they will not receive it). In contrast, when she withholds it unintentionally, it is safe to approach the reward right away (because the dogs are actually supposed to have it). In addition to waiting behaviour, we exploratively looked at the dogs’ other behavioural reactions to the (un)intentional withholding of rewards.

If dogs understand humans’ intention-in-action, they may react differentially depending on whether the experimenter intentionally or unintentionally withheld a reward and, hence, dogs will show certain reactions more in one context than another. Behavioural reactions to unintentional and intentional actions differed substantially between species in previous implementations of this paradigm. For instance, parrots opened their beak^21^ or infants reached for the object^12^. For this reason, we looked at a variety of reactions.

## Results

### Analysis of waiting

**Figure 2:**
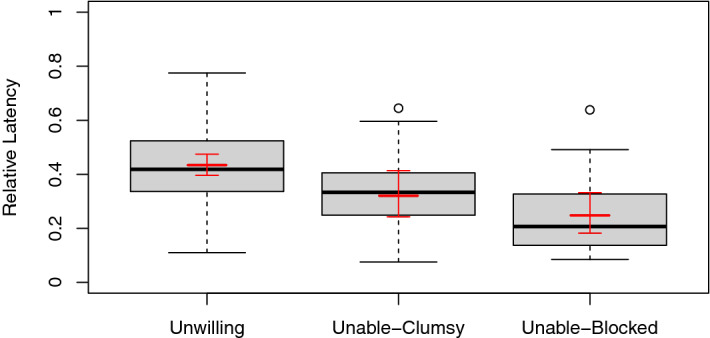
Relative latencies of going around the partition. Boxplots depict 25^th^ and 75^th^ percentiles and circles depict outliers. The fitted model is depicted in red. Error bars of the model show the 95% confidence interval.

Dogs waited longer to approach the rewards when the experimenter had withheld them intentionally than when she did so unintentionally. They also waited longer in the unable-clumsy condition than in the unable-blocked condition. Fitting a Generalized Linear Mixed Model (see Fig. 2), we looked at the effect of condition on dogs’ waiting behaviour. Waiting behaviour was operationalized by the relative latency of going around the partition: What proportion of his/her overall waiting time did the dog wait in each condition (see Method for a more detailed description). To account for repeated measures, we included random intercepts for each dog. We found an effect for condition (*χ*^*2*^ = 43.909, *df* = 2, *p* < 0.001, *R*^*2*^ = 0.609). Relative latencies were higher in the unwilling-condition than in the unable-clumsy condition (*β* ± *SE* = − 0.486 ± 0.116, *z* = − 4.176, *p* < 0.001) and in the unable-blocked condition (*β* ± *SE* = − 0.847 ± 0.121, *z* = − 7.022, *p* < 0.001). On average, a dog waited 43% of his/her overall waiting time in the unwilling-condition and only 32% in the unable-clumsy and 25% in the unable-blocked condition. We compared both unable-conditions via Tukey post hoc comparisons; relative latencies were higher in the clumsy than in the blocked condition (*β* ± *SE* = − 0.360 ± 0.123, *z* = − 2.933, *p* = 0.010, *p*-value adjusted for multiple comparisons). Adding the order of conditions, the interaction of condition and order, or dogs’ age to the full model did not improve the fit of the model (all *χ*^*2*^s ≤ 13.241, all *p*s ≥ 0.584). We also conducted separate Spearman rank correlations for each condition to test for a relation between latency and trial order. These revealed no such correlation for the unwilling or clumsy condition (all |*r*_*s*_|s ≤ 0.131, all *p*s ≥ 0.356). Only in the blocked condition, dogs tended to wait less if this condition was administered last (*r*_*s*_ = − 0.277, *p* = 0.049). To account for the fact that some dogs did not leave their basic position, we also fitted a Cox Mixed Effects Model. This yielded similar results as the Generalized Linear Mixed Model. We report these results in the Supplementary Information.

### Other behavioural reactions

**Figure 3:**
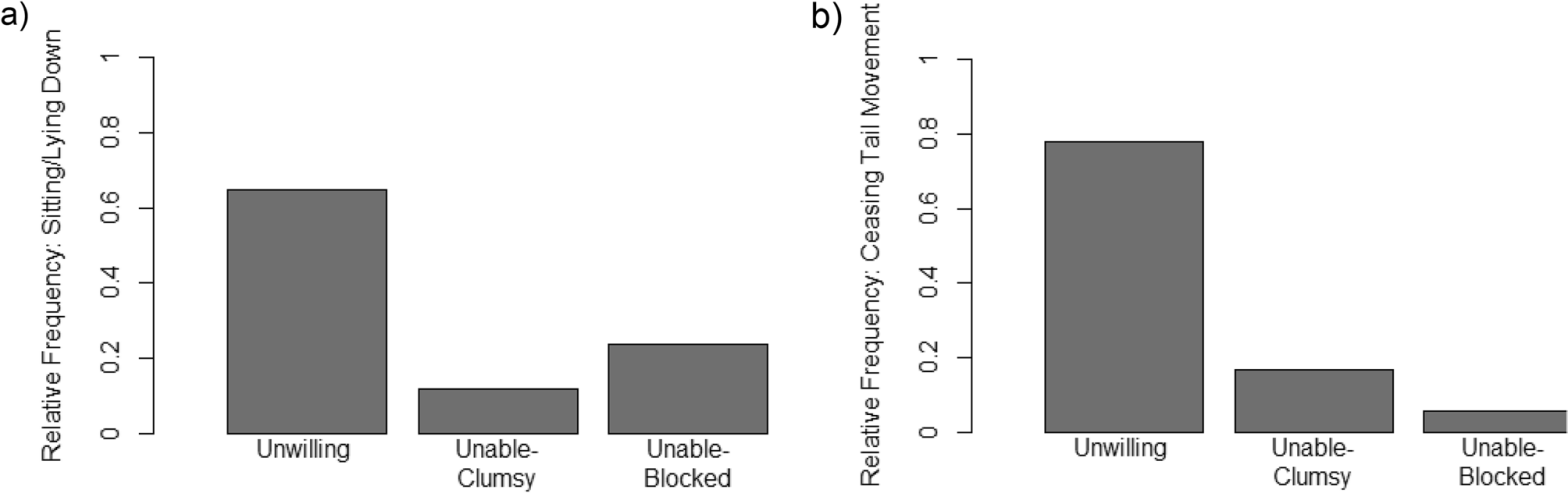
Relative frequencies of behavioural reactions. (**a**) depicts how often dogs sat/lay down relative to the number of overall occurrences of sitting and lying down. Eight dogs sat/lay down only in the unwilling condition, three dogs in the unwilling condition and at least one unable condition, and two dogs did not sit down in the unwilling-condition but only in the clumsy or the blocked condition. (**b**) Depicts the equivalent for ceasing tail movement. Eleven dogs ceased moving their tails only in the unwilling condition, three in the unwilling condition and at least one unable condition, and one dog ceased tail movement only in the clumsy condition.

This is the first time this paradigm has been adapted for dogs. For this reason, we exploratively looked at dogs’ behavioural reactions to the experimenter withholding the reward. We identified two reoccurring behaviours that occurred in frequencies that deviated between conditions: sitting or lying down and ceasing tail movement. Both behavioural reactions occurred mostly in the unwilling-condition and considerably less often in the unable-conditions (see Fig. 3). Altogether, we observed 17 occurrences of sitting or lying down among 13 dogs (three dogs showed the behaviours in more than one condition, see also Fig. 3). Of the overall occurrences, 65% (*n* = 11) were observed in the unwilling-condition, 12% (*n* = 2) in the unable-clumsy condition, and 24% (*n* = 4) in the unable-blocked condition. Two dogs did not sit down in the unwilling-condition but only in the clumsy or the blocked condition. We found a similar pattern for tail movement: Overall, ceasing tail movement occurred 18 times among 15 dogs. Of these 18 occurrences, 78% (*n* = 14) were observed in the unwilling-condition, 17% (*n *= 3) in the unable-clumsy condition, and 6% (*n* = 1) in the unable-blocked condition. One dog ceased tail movement only in the clumsy condition.

## Discussion

Dogs in our study clearly behaved differently depending on whether the actions of a human experimenter were intentional or unintentional. They waited significantly longer before approaching a reward that the experimenter had withheld intentionally than a reward that had not been administered due to human clumsiness or a physical obstacle. Thus, dogs were able to distinguish between the experimenter’s intentional and unintentional actions. This suggests that dogs may indeed be able to identify the experimenter’s intention-in-action.

Likewise, we found a difference between intentional and unintentional human action in dogs’ other behavioural reactions. The dogs that sat or lay down mainly did so in the unwilling-condition. Sitting and lying down are interpreted as so-called calming signals^53^ that are employed by dogs to appease their interaction partners. Possibly, some dogs interpreted the intentional but not the unintentional withholding as menacing or simply as confusing. While dogs might be used to their owners withholding food from them, some might have been irritated that a stranger, who readily fed them before, suddenly withdrew the reward. To defuse the situation, they might have employed sitting and lying down to appease the experimenter. Another possibility is that the withholding of the reward had an activating effect and the dogs thought that some form of learned action might convince the unwilling experimenter to supply the reward. Hence, as a best guess, they tried the commonly rewarded behaviours of sitting or lying down as they have a positive emotional predisposition which makes them more persistent and aware of signals for rewards^54^. Similarly, in other studies dogs sat down when they could not approach forbidden food as a human was watching them^24^ or when the experimenter communicated that she expected the dog to perform a certain behaviour (e.g., to open a door or to look for something) but did not provide sufficiently intelligible cues what action she expected^39,55^.

In a similar manner, the dogs that ceased to move their tail mainly did so in the unwilling-condition. Ceasing and slowing down tail movement has been interpreted as signals for attentiveness, and attempting to make sense of confusing situations^56–58^. With regard to our study, it might have confused the dogs that the experimenter suddenly started to withhold the reward intentionally. This interrupted the established pattern that the experimenter gives one reward after the other. In contrast, when she tried to administer the reward but failed, this did not interrupt this pattern and accordingly gave no reason for confusion. Thus, all three measures strongly suggest that dogs distinguished in their reactions between the experimenter’s intentional and unintentional actions. This concurs with findings of previous studies on other species that also found subjects to distinguish in their behavioural reactions to intentional and unintentional withholding of rewards^12,18–23^. Following interpretations of these findings in other species, this indicates that dogs can indeed recognize human intention-in-action.

Dogs’ behaviour in the two unable conditions may even suggest that they form different expectations regarding an actor’s persistence depending on the circumstances of her failed actions. In the case of accidents, an actor may be regarded as more likely to persevere than in the case of physical constraints blocking her success. Such differential expectations would explain why dogs tended to wait longer in the clumsy condition, but approached earlier in the blocked condition. However, this is a fairly speculative, post-hoc explanation and further research is needed to evaluate whether beyond distinguishing intentional from intentional actions, dogs make differential action predictions based on the circumstances that prevented an actor from completing her goal.

The capacity to recognize human intentional action would have been of immense value for dogs’ common history with humans. Still, our findings that they might actually have this capacity do come as an interesting surprise as a genuine understanding of intentions is generally met with a great deal of scepticism^36,59^. In line with this scepticism, interpreting dogs’ capacity to discriminate between intentional and unintentional action as a genuine understanding of intentions should be qualified by several caveats. The following part will address possible alternative explanations.

One caveat relates to special living conditions of dogs in the human environment. This is a substantial difference between species like apes, monkeys and parrots^18,19,21,22^ on the one side and dogs on the other. Dogs do not only have ample interactions with humans, but they are actively trained, also, with regards to accessing food. Accordingly, it is possible that during these interactions, dogs learn to read human behaviour in that they associate certain forms of movement or facial expressions with the appropriate reaction of approaching (or not approaching) an object^60^. Such associative behaviour rules do not allow dogs to take an intentional stance but only to rely on associations between certain cues and reactions^36^. For example, dogs might have learned that if a reward is not administered immediately, it might be administered later if they sit or lie down. Similarly, they might have learned that if a food is withdrawn, they will not receive it if they approach it. Note, however, that while it is true that dogs, in contrast to other species, experience ample of interactions with humans this is also true for infants and to a lesser extent for horses. Thus, increased interactions with humans might lead to a better discrimination between intentional and non-intentional behaviour, be it based on a genuine understanding of intentions or on associative learning of a combination of behavioural cues.

It is possible that dogs’ reactions in this study reflect some kind of socio-cognitive capacity, but not an understanding of intention-in-action. There are certain behavioural cues that in combination with these capacities might have triggered dogs’ reactions: The withholding of the reward was accompanied by different vocalizations that commonly signal intentional and accidental behaviour. The experimenter’s movements were held as identical as possible. Yet, as the experimenter was not blinded, we cannot exclude that she unconsciously provided behavioural cues. However, dogs can recognize human emotion and are good at distinguishing positive from negative emotions^61,62^. Possibly, the unwilling-condition provided more cues for negative emotions than the unable-conditions which induced dogs’ more hesitating reaction. Also, dogs might have perceived the slightly more negative intonation in the unwilling-condition as indicating a command and therefore waited longer. Alternatively, the experimenter’s behaviour might have provided cues regarding the accessibility of the reward. Withholding the rewards in the unwilling-condition might have signalled some form of possession. This might have discouraged dogs to approach the reward.

Moreover, dogs’ appropriate reactions might only reflect some form of submentalizing. For instance, dogs were rather unfamiliar with the experimenter’s behaviour and the setting. Possibly, dogs were irritated or surprised by the experimenter’s inconsistent actions in the unwilling-condition (first giving then withdrawing the reward) which was then reflected in a more hesitating reaction. This could account for the difference in dogs’ reactions between the unwilling and unable-clumsy. Yet, it cannot account for the differences between the unwilling and unable-blocked condition, as movements were identical in these conditions.

Accordingly, there are caveats concerning interpreting our findings as recognizing intentions-in-action. Note, however, that we chose rather non-ostensive vocalizations that are typical communicative signals of intention-in-action. Also, there was more similarity in the experimenter’s movements between the unwilling and unable-blocked condition than between the unable-blocked and unable-clumsy condition. Still, dogs waited longer in both unable-conditions. Thus, the movements alone cannot account for dogs’ distinguishing reactions. Moreover, the results yield a rather large effect of condition. If our findings reflected only formed (or not formed) associations, variability among dogs should have been much higher mirroring different levels of exposure to human interaction.

Nevertheless, future research needs to address these alternative explanations by systematically excluding that dogs can rely on such strategies. Such research should look at the explicit role of vocal exclamations in dogs’ reactions. To what extent do dogs use these cues for recognizing intention-in-action or something else? Also, future research should include dogs that have not had much contact with humans to control for experience with human behaviour^63^. Furthermore, it would be interesting to adapt this design to wolves. Assuming that dogs do understand human intentional action, it would be of high interest whether this capacity developed during domestication or whether it was a capacity that was already present in wolves and only had to be generalized to humans. Considering that other non-domesticated species show similar distinguishing reactions^18,19,21,22^, this seems quite possible. This would potentially explain why dogs can make sense of some but not all aspects of human intentions.

In conclusion, the present findings suggest that dogs recognize the intentionality of human action in their spontaneous behaviour. Future research needs to address whether dogs’ distinguishing reaction really reflect a capacity to read human intentions or only some form of behaviour reading based on learned associations. Nevertheless, our findings provide important initial evidence that dogs may have at least one aspect of Theory of Mind: The capacity to recognize intention-in-action.

## Method

### Ethical statement

**Figure 4:**
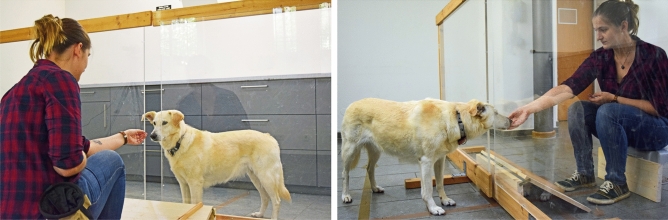
Experimental set-up.

**Figure 5:**
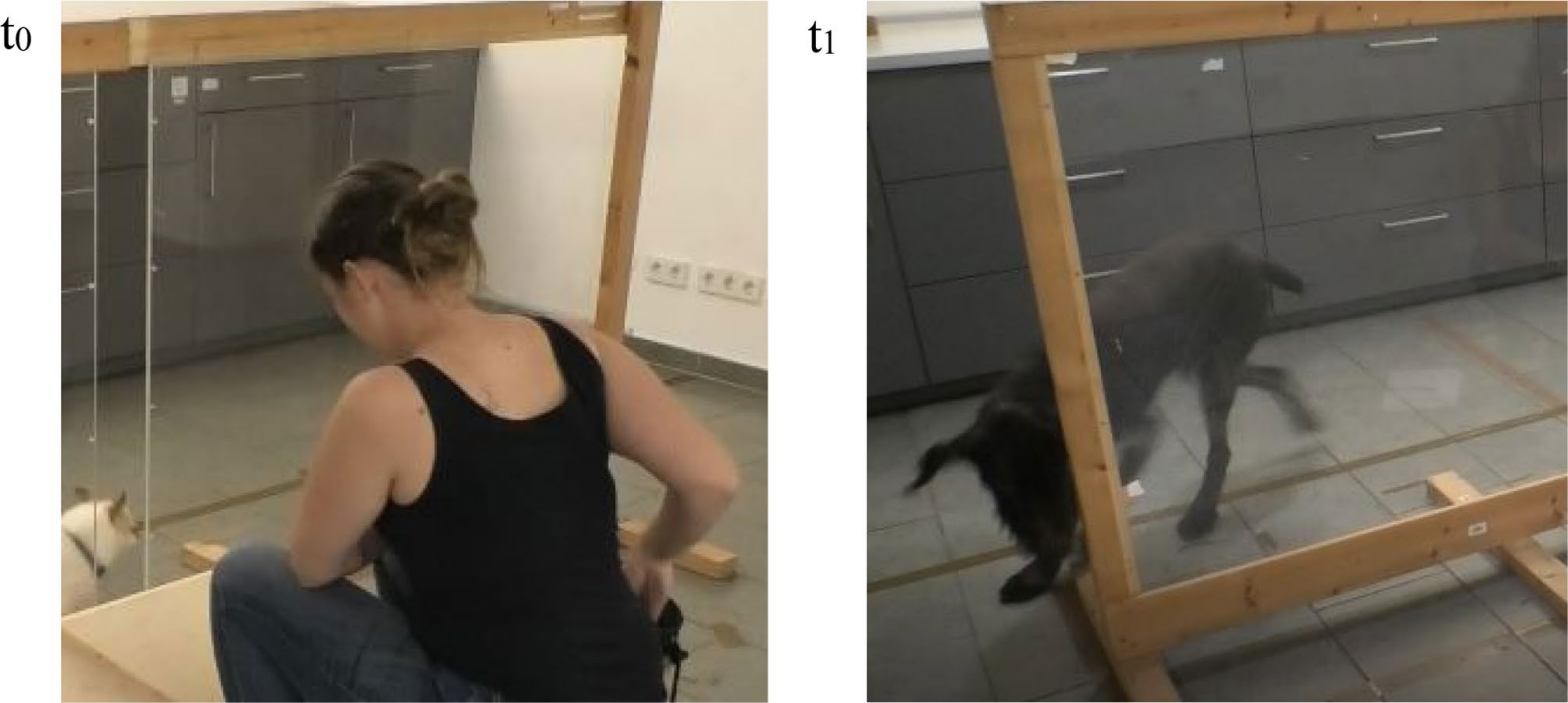
(t_0_) shows an example of a starting point of a time interval: E1 retrieves the reward. (t_1_) shows an example of an end point of a time interval: dog’s head aligns with partition.

We only tested pet dogs. All owners had given their consent prior to testing. Research was non-invasive and dogs were deprived of neither food nor water. The study was ethically approved by the Animal Welfare Body of the University of Göttingen (consultation no. E6-19). All methods were in accordance with relevant guidelines and adhered to the legal requirements of Germany. Informed consent was obtained for publication of identifying information/images (Figs. 1, 4, 5, Video S1) in an online open-access publication.

### Subjects

We analysed data of 51 dogs (27 female and 24 male) of various breeds and ages (range: 1–15 years, *M* = 5.80, *SD* = 3.05; see Supplementary for an overview of age, sex, and breed). We only tested dogs who had not received special training, as for example police dogs or registered rescue dogs. Five other dogs were tested but had to be excluded from analyses because they did not meet the inclusion criteria (see below). Sample size was estimated a priori via G*Power 3.1.9.2 assuming *η*^*2*^ = 0.2 (based on piloting results) and 1 − *ß* = 0.8. Dogs were recruited from the Dog Studies Database of the Max Planck Institute for the Science of Human History.

### Experimental set-up

Dogs were tested in a quiet room by two female experimenters (E1 and E2). The owners were not present. The apparatus (see Fig. 4) consisted of two partition walls (each wall: 1.45 m wide × 1.15 m high). Each wall consisted of a wooden frame holding a sheet of transparent plastic. One side of the frame was open. The two walls were positioned next to each other in such a way that the two open sides faced each other. They could be pushed together completely or pulled apart, to form a 15 cm-wide gap. This gap allowed the experimenter to pass rewards through the partition. It always remained open, except in the unable-blocked condition. Dogs were always placed on the opposite side of the apparatus of E1. In front of E1 was a ramp (0.35 m long × 0.65 cm wide × 0.20 m high). This ramp ensured that all rewards (whether intentionally placed or accidentally dropped) would end up approximately in the same location.

### Procedure

We allowed the dog to explore the room freely for a few minutes to familiarize him/herself with the setting. Dogs that stayed near the exit were encouraged by E1 to move around via positive verbal reinforcement.

#### Familiarization

The familiarization phase consisted of two steps. First, we showed the dogs that they could go around the partition. E1 directed the dog to the basic feeding position facing the partition wall. E1 herself took place on the opposite side of the partition. To test whether the dog understood that they could go around the separation wall on either side, E1 encouraged the dog verbally to go around the partition and approach her. E1 stayed seated during this process. Only when dogs failed to go around the partition, E1 went halfway around the wall to demonstrate this possibility. This was repeated until the dog successfully went around the wall. Dogs who did not reach this criterion within 1.5 min were excluded (*n *= 2). Afterwards, E1 redirected the dog back to the basic feeding position. In the second step, the dogs were familiarized with the administration of rewards through the gap in the partition. E1 administered rewards through the gap. Dogs were required to take five rewards in a row without leaving their basic position. We excluded dogs that did not reach this criterion (e.g., because of distraction, *n* = 3). Only then was the test phase started.

#### Test

Each dog received one test trial per condition in a balanced order. In between test trials, we administered filler trials.

#### Test trials

The rationale of each condition was as follows: E1 tried to give a reward to the dog but, for either intentional or unintentional reasons, ended up not doing so. Instead, either she placed the reward immediately in front of herself on the floor or the reward fell out of her hand. The movement of (not) giving the reward was repeated up to five times. These movements were identical in terms of pace (roughly 23 s) throughout the conditions. Then, E1 turned away and focused on her coding sheet for five seconds. When the dog went around the partition before all five rewards were placed on the floor, E1 terminated the trial. Each condition’s presentation took roughly 28 s, unless dogs left the basic position earlier.

#### Unwilling-condition

The gap between the two walls was open. E1 moved a reward towards the gap but intentionally pulled the reward away in a fast motion. While pulling it away, she exclaimed “ha-ha!”. These utterances were added as non-ostensive but common vocal communicative signals that are used to underline an attitude towards actions. E1 then placed the reward immediately in front of herself, on her side of the partition.

#### Unable-clumsy condition

This condition was similar to the unwilling-condition, but with one exception: Instead of intentionally pulling the reward away, E1 “accidently” dropped the reward and exclaimed “oops!”. The rewards were dropped onto the ramp. This way, they rolled to the same location where E1 put them in the other conditions.

#### Unable-blocked condition

In this condition, E2 entered the room and closed the gap between the partition walls by pushing both walls together. E1 then moved the reward towards the wall, but the partition made it impossible to administer the reward. When hindered, she exclaimed “oh!” and put the reward in front of her. The speed and nature of this movement was similar to the unwilling-condition. Note that some dogs reacted to the closing of the gaps. Dogs however immediately reoriented their attention, when the first reward was held up. Twelve dogs went around the walls as a reaction to closing the partition walls. In these cases, we repeated the familiarization and closed the gap a second time.

#### Filler trials

In the filler trials, E1 gave rewards to the dog through the gap. Just as in the familiarization phase, this was done until the dog had eaten five rewards in succession without leaving the basic position. The reason behind this was to maintain “E1 gives rewards through the gap” as the default event.

### Coding

All test sessions were recorded. Videos were then used for coding. We used BORIS^64^ to code the dogs’ behaviour. To analyse dogs’ waiting behaviour, we coded the latencies of going around the partition. Absolute latencies were coded as the time interval t_0_ – t_1_:

t_0_: E1’s hand leaves the bag of rewards (retrieving the reward).

t_1_: Dog’s head aligns with partition wall (see Fig. 5 for an example).

To account for inter-individual differences, we computed the dogs’ relative latencies: For each dog, we divided his or her latency in one condition by the sum of his or her latencies in all three conditions.

Moreover, we coded dogs’ behavioural reactions that occurred in the course of this time interval (t_0_ – t_1_; for a full ethogram see Supplementary Material). Latencies and behavioural reactions were coded by two second coders who were blind to hypotheses. They coded 20% of the subjects. Interrater agreement for both measures was almost perfect (latencies: (*r*_*s*_ = 0.975), behavioural reactions: *κ* = 0.982).

## Supplementary Information

Supplementary Information.

Supplementary Video.
