# Supplementary material for: Dogs distinguish human intentional and unintentional action
Source: Sci Rep. 2021 Sep 1;11:14967. doi: 10.1038/s41598-021-94374-3 (PMC8410798; doi:10.1038/s41598-021-94374-3)
Supplement: Supplementary file 1 — Supplementary Information. [file 41598_2021_94374_MOESM1_ESM.pdf]

Dogs distinguish human intentional and unintentional action

Supplementary Material

Britta Schünemann[1]*, Judith Keller[1]*, Hannes Rakoczy[1], Tanya Behne[1], & Juliane Bräuer[2,3]

[1]*Department of Developmental Psychology, University of Göttingen, Waldweg 26, 37073 Göttingen, Germany*

[2]*Max Planck Institute for the Science of Human History, Department of Linguistic and Cultural Evolution, Kahlaische Strasse 10, 07745 Jena, Germany*

[3]*Department for General Psychology and Cognitive Neuroscience Friedrich Schiller University of Jena, Am Steiger 3, 07743 Jena, Germany*

* Shared first authorship

Address for correspondence: Britta Schünemann, Georg-August-Universität Göttingen, Waldweg 26, 37073 Göttingen, Germany. Email: britta.schuenemann@uni-goettingen.de
ORCID iD Britta Schünemann: 0000-0002-7493-5904

Supplementary Materials

Analysis of Waiting. To analyse whether dogs waited longer in the unwilling than in the unable conditions, we fitted a Generalized Linear Mixed Model with beta error distribution and logit link function. We included Condition as a fixed effect in the model. Condition was dummy coded with unwilling being the reference level. To account for repeated measures, we included dogs' ID as random intercept effect. The dependent variable, waiting behaviour, was operationalized by the relative latency of going around the partition (latency of condition divided by sum of latencies in all three conditions). The model was stable (see Fig. S1) and not overdispersed (dispersion parameter=1.038). Based on a likelihood ratio test, we compared this full model to a null model including only dogs' ID as random intercept effect.

full model: Relative Latency ~Condition+ (1| Dog's ID), family= beta, link=logit

null model: Relative Latency ~ (1| Dog's ID), family= beta, link=logit

The full-null model comparison revealed a main effect for Condition ($\chi 2$=43.909, *df*=2, *p*<.001, AIC (full model)= -183.681). See Table S1 for the detailed coefficients of the full model. Adding age to the full model did not increase the fit of the model ($\chi 2$=0.0139, *df*=1, *p*=0.906, AIC = -181.695). This was also the case for adding order of condition ($\chi 2$=0.201, *df*=5, *p*=.999, AIC=-173.882) or the interaction of order and condition ($\chi 2$=13.241, *df*=15, *p*=.584, AIC = -166.922).

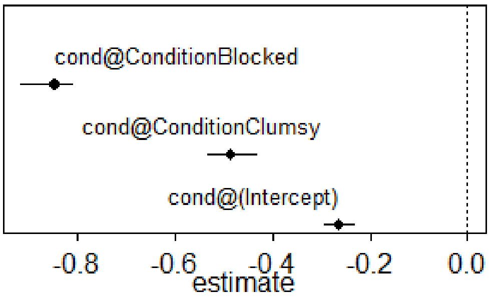

Figure S1. To determine model stability, one level of the random effect (Dog's ID) was dropped at a time. The figure shows the range of obtained estimates.

Table S1. Results of the full model with unwilling as reference level

term	*β*	*SE*	lower CI	upper CI[2]	*z*	*p*
intercept	-0.264	0.080	-0.428	-0.107	-3.293	0.001
condition unable-clumsy[1]	-0.486	0.116	-0.718	-0.241	-4.176	0.000
condition unable-blocked[1]	-0.847	0.121	-1.083	-0.606	-7.022	0.000

[1]condition was dummy coded with unwilling being the reference level
[2]confidence were obtained via parametric bootstrap

Some dogs did not leave the basic feeding position at all. This was coded as the maximum latency of 27 seconds. To account for this, we also fitted a Cox mixed effects model on the raw measure of latency. This model accounts for individual baseline differences as well as time intervals where no t_1 could be collected. As before, we included the condition as a fixed effect in our model and Dog's ID as random intercept in our full model. The dependent variable was latency accounting for trials without determined t_1. Again, this full model was compared to a null model including only the random intercept. A comparison of full and null model revealed a significant effect of condition (χ^2=33.894, *df*=2, *p*<.001). Dogs waited longer in the unwilling condition than in both unable conditions (see Table S2). Adding order to the full model did not increase the fit of the model (χ^2=10.598, *df*=5, *p*=.060).

Table S2. Results of the full cox model

term	*β*	*SE*	*z*	*p*
condition unable-clumsy[1]	0.936	0.250	3.74	<.001
condition unable-blocked[1]	1.481	0.253	5.85	<.001

[1]condition was dummy coded with unwilling being the reference level

We found similar results fitting a conducting a non-parametric Friedman's ANOVA and fitting a Bayesian Generalized Linear Mixed Model. Friedman's ANOVA revealed a main effect on relative latencies for Condition (χ^2=28.138, *df*=2, *p*<.001). Post hoc tests with Bonferroni correction applied indicated that dogs waited significantly longer in the unwilling condition than in both unable conditions (clumsy: observed difference= 28.0, *p*=.015; blocked: observed difference=51.5, *p*<.001; critical difference: 24.178). We fitted a Bayesian Generalized Linear Mixed Model with beta error distribution and logit link and included the same effects model as in our main frequentist analysis. Again, we found that dogs waited longer in the unwilling condition than in the unable-clumsy condition (*β*=-0.49, 95% CI [−0.73, −0.26]) and in the unable-blocked condition (*β*=-0.85, 95% CI [−1.08, −0.61]).

We used the following software and packages for analyses[8–21].:

- RStudio Version 1.1.463 and Version 1.3.1093 (for Bayesian modelling)

- brms (2.14.4), car (3.0-6), coxme (2.2-16), dplyr (1.0.5), glmmTMB (1.0.1), lme4 (1.1-21), multcomp (3.2-11), pgirmess (1.7.0), reshape2 (1.4.3), rstan (2.21.2), shinystan (2.5.0), survival (3.2-11), tidybayes (2.3.1), performance (0.7.1)
- boot.glmmTMB and glmmTMB_stability function provided by Roger Mundry (2020)

Table S2. Overview of behavioural reactions in previous studies using the Unwilling vs Unable Paradigm[1–7].

Animal	Measures	Conditions	Findings	Source
Chimpanzees	Latency till leaving testing area<br>Knocking on glass<br>Poking finger in hole<br>Moving apparatus	*3 Unwilling:*<br>*Refuse, Eat, Tease*	increased behavioural rate	Call et al., 2004
		5 Unable:<br>*Clumsy, Blocked, Distracted, Search, Blind*	Stayed longer in testing area	
Human Children	Reaching behaviour<br>Spontaneous gestures<br>Banging on the table<br>Time spent looking at experimenter<br>(Positive/negative affect)	*3 Unwilling:*<br>*Refuse, Tease, Play, (Mock)*	Increased reaching for toy<br>Increased spontaneous gestures<br>Increased banging on the table<br>(Increased negative affect)	Behne et al., 2005, (Marsh et al., 2010)
		6 Unable:<br>*Clumsy, Reach, Open, Talk, Telephone, Search, (Blocked)*	Longer time spent looking at experimenter<br>(Increased positive affect).	
Capuchin Monkeys	Latency till leaving testing area	*1 Unwilling:*<br>*Tease*		Phillips et al., 2009
		2 Unable:<br>*Clumsy, Blocked*	Stayed longer in testing area	
Grey Parrots	Time facing away<br>Vocalisation<br>Wire mesh biting<br>Table scraping/ Knocking	*1 Unwilling:*<br>*Tease*	Increased beak opening<br>Increased table scraping	Peron et al., 2010
		2 Unable:<br>*Distracted, Blocked*	Increased wire biting	
Tokean Macaques	Latency till leaving testing area<br>Gazing alterations<br>Aggressive Behaviour<br>Begging<br>Spontaneous Gestures	*1 Unwilling:*<br>*Tease*	Increased gaze alteration frequency<br>Increased aggressive behaviour	Canteloup & Meunier 2017
		2 Unable:<br>*Clumsy, Distracted*	Increased begging<br>Increased spontaneous gestures	
Horses	Percentage of time before facing away<br>Percentage of time touching barrier	*1 Unwilling:*<br>*Tease*		Trösch et al. 2020
		2 Unable:<br>*Clumsy, Blocked*	Higher Percentage of time before facing away<br>Higher percentage of time touching barrier	

Table S3: Ethogram of behaviours that have been coded in the time interval t_0 - t_1 for each condition (adapted from Protopopova and colleagues[22]).

Behaviour	Definition	Unwilling	Clumsy	Blocked	Total
**Position**					
Standing	Supported upright with all four legs (starting position)				
Lying	Lying down with limbs either tucked under or placed in front of body	3	0	0	3
Sitting	Supported by two extended front legs and two flexed back legs	8	3	4	15
Beg position	At least one front paw lifted of ground while the back legs remain flexed	2	3	4	9
**Face**					
Facing forward	Head is oriented so that it is facing the experimenter (starting position)				
Facing away	Head is oriented so that the experimenter only sees the profile of the dog	8	2	11	21
**Locomotion**					
Standing up	Change of position from sitting or lying into standing	7	1	2	9
Moving away	Dog moves away from the separation wall/experimenter	0	0	0	0
Moving forward	Dog moves around the separation wall in direction to the treat	40	45	47	132
**Vocalization**					
Barking	Vocalization of very short duration and low frequency	2	0	1	3
Growling	Throaty, rumbling vocalization	0	0	0	0
**Maintenance**					
Yawning	Opens mouth widely and inhales	0	0	0	0
Panting	Tongue exposed with audible and/or observable breathing	9	9	10	28
Scratching	Paw makes repeated contact with body; head may be angled in direction of moving limb	0	0	1	1
**Tail position**					
Wagging tail	Tail moves perpendicular to the dog’s body	29	28	30	87
Stop Wagging	Ceasing of tail wagging at the start of/during the condition	14	3	1	18

Table S4. List of the tested dogs with age, sex, and breed.

Dog-ID	Age	Sex	Breed	Mix: Known Breeds
203	6	f	Border Collie	
106	10	m	Chihuahua	
177	3	m	Eurasian	
230	3	m	Fox Terrier	
158	5	f	French Bulldog	
220	1	m	French Bulldog	
227	3	f	French Bulldog	
228	7	f	Galgo Espanol	
95	5	m	German Shepherd	
213	2	m	German wire-haired Pointer	
20	9	m	Golden Retriever	
33	10	m	Golden Retriever	
159	3	m	Golden Retriever	
193	4	m	Golden Retriever	
202	2	f	Golden Retriever	
224	3	m	Golden Retriever	
197	8	m	Irish setter	
180	4	f	Jack Russel Terrier	
195	4	m	Labrador	
225	1	f	Malinois	
78	13	f	Mix (big)	English Pointer/ Podenco
46	7	m	Mix (big)	
113	9	f	Mix (big)	Bernese Mountain Dog
200	10	f	Mix (big)	Canadian Eskimo Dog
205	5	f	Mix (big)	German Shepherd
214	8	m	Mix (big)	Bernese Mountain Dog/ Old German Herding Dog
226	3	m	Mix (big)	Boxer
111	8	m	Mix (midsize)	Labrador
156	4	f	Mix (midsize)	German Shepherd/Rottweiler
188	4	f	Mix (midsize)	Golden Retriever
199	8	m	Mix (midsize)	German Shepherd/Rottweiler
215	1	f	Mix (midsize)	Old German Herding Dog
223	15	f	Mix (midsize)	Border Collie
234	4	f	Mix (midsize)	Australian Shepherd/ Beagle
98	9	f	Mix (small)	
99	8	f	Mix (small)	
100	8	f	Mix (small)	
162	3	m	Mix (small)	Bolonka Zwitna
163	6	m	Mix (small)	Bolonka Zwitna
194	8	m	Mix (small)	Beagle/ Jack Russel Terrier
198	1	m	Mix (small)	
170	3	f	Pug	
171	3	m	Pug	
83	6	f	Ratonero Bodeguero Andaluz	
30	3	m	Rottweiler	
79	6	f	Small Munsterlander	
221	12	f	Small Munsterlander	
4	12	f	Vizsla	
231	1	f	Vizsla	
229	1	f	White Swiss Shepherd	
232	4	f	Wire-haired Dachshund	

Table S5: Orders of conditions W = Unwilling, B= Unable-blocked, C=Unable-clumsy and the number of dogs that have been tested in this order.

Order	N
WCB	9
WBC	8
BWC	9
BCW	8
CBW	9
CWB	8

References

1. Trösch, M., Bertin, E., Calandreau, L., Nowak, R. & Lansade, L. Unwilling or willing but unable: can horses interpret human actions as goal directed? *Anim. Cogn.* **23**, 1035–1040 (2020).

2. Phillips, W., Barnes, J. L., Mahajan, N., Yamaguchi, M. & Santos, L. R. 'Unwilling' versus 'unable': capuchin monkeys' (*Cebus apella*) understanding of human intentional action: Unwilling vs. unable in capuchin monkeys. *Dev. Sci.* **12**, 938–945 (2009).

3. Péron, F., Rat-Fischer, L., Nagle, L. & Bovet, D. 'Unwilling'versus 'unable': Do grey parrots understand human intentional actions? *Interact. Stud.* **11**, 428–441 (2010).

4. Marsh, H. L., Stavropoulos, J., Nienhuis, T. & Legerstee, M. Six- and 9-Month-Old infants discriminate between goals despite similar action patterns. *Infancy* **15**, 94–106 (2010).

5. Canteloup, C. & Meunier, H. 'Unwilling' versus 'unable': Tonkean macaques' understanding of human goal-directed actions. *PeerJ* **5**, e3227 (2017).

6. Call, J., Hare, B., Carpenter, M. & Tomasello, M. 'Unwilling' versus 'unable': chimpanzees' understanding of human intentional action. *Dev. Sci.* **7**, 488–498 (2004).

7. Behne, T., Carpenter, M., Call, J. & Tomasello, M. Unwilling versus unable: infants' understanding of intentional action. *Dev. Psychol.* **41**, 328–337 (2005).

8. Lüdecke, D., Ben Shachar, M. S., Patil, I., Waggoner, P. & Makowski, D. *Assessment, testing and comparison of statistical models using R. R package version 0.7.1*. (2021).

9. Kay, M. *tidybayes: tidy data and geoms for bayesian models. R package version 2.3.1*. (2020).

10. Therneau, T. M. *A package for survival analysis in R. R package version 3.2-11.*

(2021).

11. Wickham, H., Francois, R., Henry, L. & Müller, K. *dplyr: a grammar of data manipulation. R package version 1.0.5*. (2021).

12. Wickham, H. *Reshaping data with the reshape package. R package version 1.4.3*. (2007).

13. Therneau, T. M. *coxme: Mixed effects cox models. R package version 2.2-16*. (2020).

14. Stan Development Team. *RStan: the R interface to stan. R package version 2.21.2*. (2020).

15. Hothorn, T., Bretz, F. & Westfall, P. *Simultaneous inference in general parametric models. R package version 3.2-11*. (2008).

16. Giraudoux, P. *pgirmess: Spatial analysis and data mining for field ecologists. R package version 1.7.0*. (2021).

17. Gabry, J. *shinystan: Interactive visual and numerical diagnostics and posterior analysis for bayesian models. R package version 2.5.0*. (2018).

18. Fox, J. & Weisberg, S. *An {R} companion to applied regression. R package version 3.0-6*. (2019).

19. Bürkner, P. C. *Advanced bayesian multilevel modeling with the R package brms. R package version 2.14.4*. (2018).

20. Brooks, M. E. *et al. glmmTMB balances speed and flexibility among packages for zero-inflated generalized linear mixed modeling. R package version 1.0.1*. (2017).

21. Bates, D., Maechler, M., Bolker, B. & Walker, S. *Fitting linear mixed-effects models using lme4. R package version 1.1-21*. (2015).

22. Protopopova, A., Mehrkam, L. R., Boggess, M. M. & Wynne, C. D. L. In-kennel behavior predicts length of stay in shelter dogs. *PloS One* **9**, e114319 (2014).
